# The development and validation of a digital biomarker for remote assessment of Alzheimer's diseases risk

**DOI:** 10.1177/20552076241228416

**Published:** 2024-01-23

**Authors:** Joe Butler, Tamlyn J Watermeyer, Ellie Matterson, Emily G Harper, Mario Parra-Rodriguez

**Affiliations:** 1 Faculty of Health Sciences and Wellbeing, Helen McArdle Nursing and Care Research Institute, University of Sunderland, Sunderland, UK; 2 Faculty of Health and Wellbeing, School of Psychology, University of Sunderland, Sunderland, UK; 3 Faculty of Psychology, University of Anahuac Mexico, Mexico City, Mexico; 4 Department of Psychology, Faculty of Health & Life Sciences, 5995 Northumbria University, Newcastle, UK; 5 Edinburgh Dementia Prevention, Centre for Clinical Brain Sciences, College of Medicine and Veterinary Sciences, University of Edinburgh, Edinburgh, Scotland, UK; 6 Community Mental Health for Older People Team, Tees Esk & Wear NHS Foundation Trust, Durham, England, UK; 7 School of Psychological Sciences and Health, 3527 University of Strathclyde, Glasgow, UK

**Keywords:** Digital biomarker, Alzheimer's disease, remote cognitive testing, remote monitoring, tele-medicine, tele-neuropsychology, web-based testing

## Abstract

**Background:**

Digital cognitive assessment is becoming increasingly widespread in ageing research and care, especially since the COVID-19 pandemic. Remote online collection provides opportunities for ageing and dementia professionals to collect larger datasets, increase the diversity of research participants and patients and offer cost-effective screening and monitoring methods for clinical practice and trials. However, the reliability of self-administered at-home tests compared to their lab-based counterparts often goes unexamined, compromising the validity of adopting such measures.

**Objective:**

Our aim is to validate a self-administered web-based version of the visual short-term memory binding task (VSTMBT), a potential digital biomarker sensitive to Alzheimer's disease processes, suitable for use on personal devices.

**Methods:**

A final cross-sectional sample of 37 older-adult (51–77 years) participants without dementia completed our novel self-administered version of the VSTMBT, both at home on a personal device and in the lab, under researcher-controlled conditions.

**Results:**

ANOVA and Bayesian *t*-test found no significant differences between the task when it was remotely self-administered by participants at home compared to when it was taken under controlled lab conditions.

**Conclusions:**

These results indicate the VSTMBT can provide reliable data when self-administered at-home using an online version of the task and on a personal device. This finding has important implications for remote screening and monitoring practices of older adults, as well as supporting clinical practices serving diverse patient communities. Future work will assess remote administration in older adults with cognitive impairment and diverse socio-economic and ethno-cultural backgrounds as well as a bench-to-bedside application.

## Introduction

Detecting the earliest stages of Alzheimer's disease (AD) and dementia thorough sensitive cognitive testing is a growing priority within dementia research and care. The COVID-19 crisis caused widespread disruption to dementia research and clinical practice, with assessment and care activities continuing in part, or fully, through remote administration. As a result, the future of ageing and dementia research has been fundamentally changed through the adoption of digital-based assessments. Prior to the pandemic, initial reports from some research programmes provided preliminary support for the use of web-based cognitive measures to enhance screening and recruitment efficiency for clinical trials as well as prospectively monitor middle-aged and older adults at risk of cognitive decline,^1–3^ thereby mitigating concerns regarding scalability and participant accessibility, engagement and retention. However, for several measures used across these registries their validation with in-person clinic- or lab-based counterparts remains forthcoming, raising concerns within the psychometric community regarding their widespread adoption during and since the pandemic.^4^

The visual short-term memory binding task (VSTMBT) is a promising cognitive measure^5^ for the identification of AD-specific dementia, as well as the prodromal and preclinical stages of AD, as demonstrated in a number of laboratory-based studies.^6–9^ Unlike traditional cognitive measures, the performance on the VSTMBT has been shown to be insensitive to age,^10–13^ education and literacy^14^ and shows not only high sensitivity but also high specificity for AD.^9,15–17^

Online data collection using the VSTMBT offers the opportunity to develop a novel digital AD biomarker, run large-sampled studies and increase the diversity of research participants by facilitating participation from under-represented groups.^18^ On the other hand, despite data indicating that the assessment properties of the VSTMBT remain reliable across traditional mediums, such as computer, tablets, and printed flashcards^19^ with administered by a researcher, no version of the task suitable for online, remote self-assessment has been compared with data collected under more stringent lab-based conditions. Our aim is to validate a web-based version of the VSTMBT that is suitable for remote, self-administration on personal devices.

## Methods

We set out to validate a web-based, self-administered version of the VSTMBT and compare lab-based performance with home-based performance. A power analysis using G*Power 3^20^ was carried to determine the sample required to detect a medium effect size (partial eta squared = 0.06, *f* = 0. 0.25) with a power ≥ 0.80 and an alpha = 0.05 in a repeated-measures design with one group and two measurements (home vs. lab). A total of 33 participants would be required to reach 80.4% chance of correctly rejecting the null hypothesis of no differences between testing locations.

Regardless of whether the participant's first testing session was lab- or home-based, they received an Information Sheet and an opportunity to discuss their participation with the research team. Once they had indicated they wanted to take part, all participants were directed to the online platform where a copy of the Information Sheet was made available along with a digital consent form. Participants did not proceed with the study unless they indicated their consent to do so. Initially, 47 participants were recruited via email from a community research registry of 699 older adults (>49 years) between September 2021 until October 2022, during relaxed North-East England, UK pandemic social restrictions. However, due to personal device failures (*n* = 4), failure to attend lab-based assessment following at-home assessment (*n* = 3) and failure to complete at-home assessment following lab-based assessment (*n* = 3), a remaining 37 participants were included in the current study. These were older adults (72.97% female; 100% white) with an age range of 51–77 years (mean age 65.89 years).

Participants were excluded if they reported a history of visual disturbances, major dexterity issues and cognitive and/or neurodevelopmental disorders using self-report (see Supplementary Materials for screening questions). Furthermore, a screen for perceptual binding problems which might account for short-term binding problems was embedded within the online cognitive task (at the start of the task); no participants failed this screen (accuracy score < 8/10 screen trials). Eligible participants answered demographic questions about age, sex, and years of education, before completing the VSTMBT. Furthermore, in the home-testing condition, participants completed the Computer Anxiety Scale^21^ and some additional questions about their home environment (see Supporting Information). In the lab-testing condition, a researcher also administered the Addenbrooke's Cognitive Examination-III (ACE-III). One participant completed the mini-Addenbrooke's Cognitive Examination (M-ACE)^22^ due to time pressure. Evidence suggests that scores on M-ACE and ACE-III are comparable.^23^ Participants completed the same testing protocol both at home and in the lab (the order was counterbalanced, participants were randomly assigned to one of these conditions) 48-hour apart (one participant was unable to meet this deadline) and there was no stipulation to take the task at the same time of day. All questionnaires and tasks, aside from the ACE-III, were programmed and administered in Psytoolkit^24,25^ and required a computer/laptop with a keyboard and mouse to complete.

### Ethics statement

Ethical approval was granted by the University of Northumbria at Newcastle Ethics Board (Reference No: 31372).

### Cognitive task

**Figure 1.**
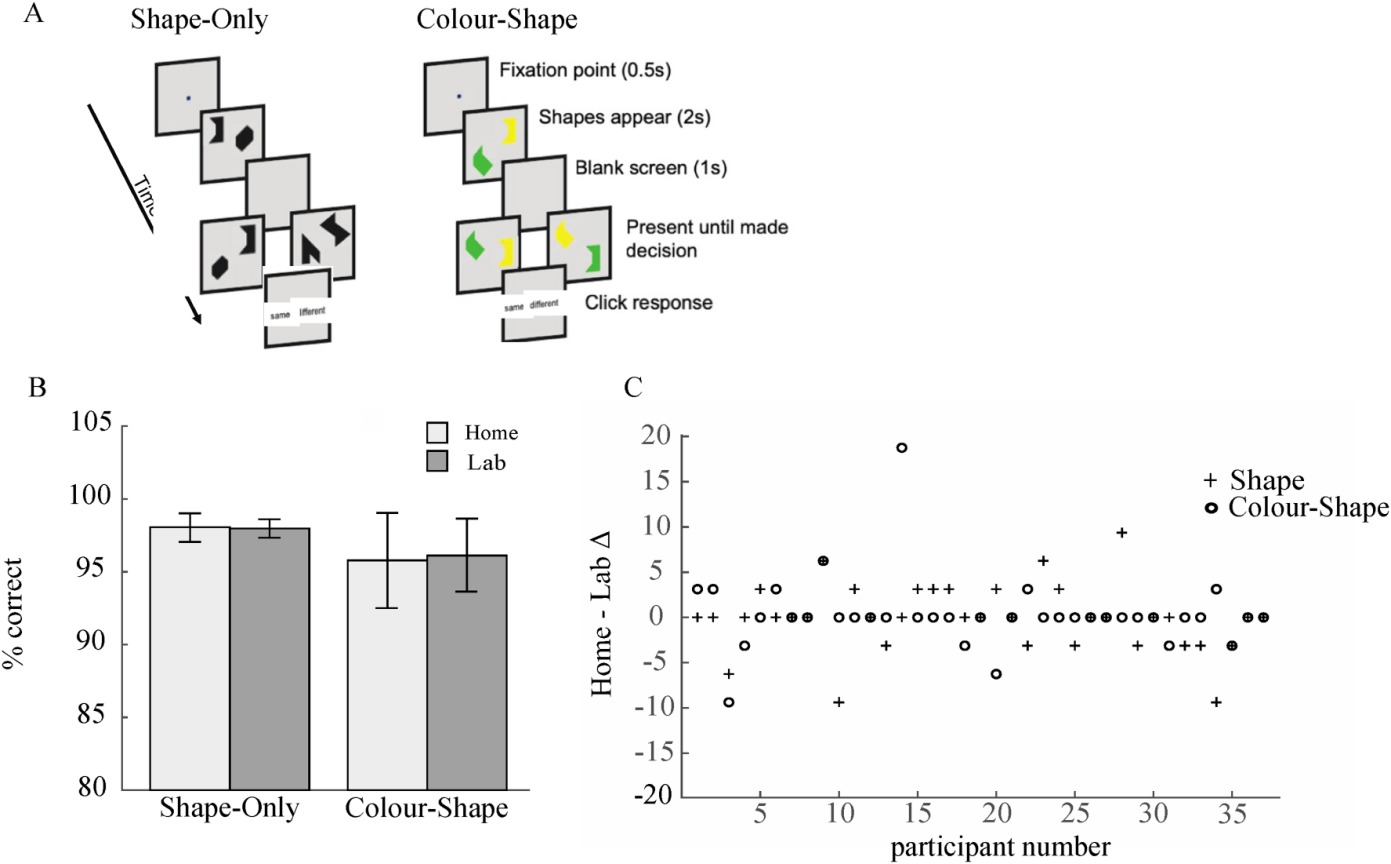
(a) The two conditions used in this current version of the task. In both conditions participants are shown a fixation point for 0.5 seconds, followed by two shapes for 2 seconds, followed by a blank screen for 1 second, followed by a further two shapes for 2 seconds. Participants must decide if the second presentation of shapes are the same as the first presentation of shapes. In the shape only condition, the second presentation of shapes is different for half of the 32 trials and the same for the remainder. In the colour-shape condition, the colours are swapped between the shapes for half of the 32 trials and the same for the remainder (b) Group means for the shape-only and colour-shape condition by testing location (home vs. lab). (c) Home - Lab difference scores for shape and colour-shape condition for each individual participant.

We used the task with settings suggested by Parra^8^ which uses the shape-only and colour-shape conditions (see Figure 1(a) for a schematic illustration). Each of the two conditions comprised of 32 trials with 16 same trials and 16 different trials. In each condition, participants were shown two objects for 2 seconds, participants were then shown a blank screen for 1 second, followed by the presentation of two further shapes. Participants had to state if the second presentation of shapes were the same or different to the first presentation of shapes. In the shape-only condition, the shapes were black and in the 16 different trials, the shapes were different in the second presentation. In the coloured-shape condition, each of the two shapes was a different colour, on different trials the colours were swapped between shapes. The binding task was preceded by a perceptual screen to ensure that binding errors were not an artefact of perceptual binding deficits. Specifically, participants had to identify if the three coloured shapes presented above a line were the same as the coloured shapes below the line. The perceptual screen comprised of 10 trials with 5 same trials and 5 different trials. On different trials, the colours were swapped between two of the shapes below the line.

Instructions were provided to participants both in written form and a video-demonstration with the opportunity to repeat instructions if they were unclear (all participants watched the instructions at least once to proceed but could rewatch these as many times as required). Participants were also given the opportunity to contact a member of the research team if they wanted further information (no participants contacted the research team) on how to complete this task. Additionally, prior to completing each of the two conditions in the binding task, participants completed four training trials (with the option of repeating if required, although no participants repeated these more than once). No feedback was provided to participants about their performance during training trials.

### Statistical analysis

We first analysed the data using a repeated measures ANOVA with two factors on individual accuracy scores. The first factor was condition (shape vs. colour-shape). The second factor was ‘testing location’ (home vs. lab). Following this, we conducted a Bayesian one-sampled *t*-test on the difference scores between lab and home performance for the shape and colour conditions. These scores were derived by subtracting each individual participant's accuracy scores in the home condition from their score in the lab condition (see Figure 1(c)). The Bayesian one-sample *t*-test was carried out separately for the shape and colour-shape conditions. In our Bayesian analysis, the main model posited that the change scores would differ from zero, while the comparative model assumed that the scores would not significantly differ from zero. Unlike traditional frequentist statistics, which aims to reject the null hypothesis, Bayesian analysis allows us to directly weigh the evidence for the null hypothesis (there is no difference between testing locations) against the main hypothesis (there is a difference between testing locations).^26^ Both the frequentist and the Bayesian analysis were conducted using JASP version 0.12.2.^27^

## Results

### Sample characteristics

Participant characteristics, average performance on the ACE-111 and scores on the Computer Anxiety Rating Scale (CAR) are shown in Table 1.

**Table 1.** Sample characteristics.

	Mean (SD) [Range]
Age	65.89 (6.83) [51–77]
Years of education	16.16 (6.08) [10–44]
ACE-III total score^a^, 0–100	92.08 (4.50) 81–100]
Attention, 0–18	17.02 (1.30) [12–18]
Memory, 0–26	23.61 (2.72) [16–26]
Fluency, 0–14	12.31 (1.62) [8–14]
Language, 0–26	25.61.61 (0.73) [23–26]
Visuospatial, 0–26	13.53 (1.50) [9–16]
Computer Anxiety Rating Scale (CAR), 19–95	41.22 (13.98) [21–76]

Participant table describe age (*n* = 37), years in education (*n* = 37), ACE-III total score and ACE-III sub-categories (higher is better on the main scale and all subscales) (*n* = 36), and the CAR scale (higher the score, higher the level of computer anxiety) (*n* = 37).

^a^ Note: one participant received the M-ACE rather than the ACE-III due to time constraints; performance was as follows: Total 26/30; Attention 3/4; Memory 14/14; Fluency 6/7; Visuospatial 3/5.

### ANOVA

We ran repeated measures ANOVA with factors of ‘testing location’ (home vs. lab) and ‘condition’ (shape vs. colour-shape) on individual participant accuracy scores (see Figure 1(b) for results). We found no significant effect of Condition *F*(1,36) = 1.640, *p* = .208, *n*^2^ = 0.038. Crucially, there was no significant effect of Location *F*(1,36) = 0.070, *p* = .793, *n*^2^ = 0.0001415 and no location by condition interaction *F*(1,36) = 0.233, *p* = .632, *n*^2^ = 0.0003931.

### Bayesian *t*-test

To confirm the null result, we next calculated difference scores for each participant and each condition by subtracting accuracy scores measured in the lab from accuracy scores measured at home (see Figure 1(c) for individual participant difference scores). We analysed this using a Bayesian one sample t-test with the main hypothesis that the test value would be significantly different from 0 and the alternative hypothesis that change scores would not be significantly different from 0. The Cauchy prior was 0.707. The results for the shape condition (BF_01_ = 5.608, error% = 0.046) provides ‘moderate’^28^ support for the null and indicates the results are 5.6 times more likely to occur under the null hypothesis (no difference between testing environment scores) than the main hypothesis (there is a difference). The results for the colour-shape condition (BF_01_ = 5.024, error% = 0.046) indicates ‘moderate’^28^ support for the null and demonstrates that the data are 5 times more likely to occur under the null hypothesis.

### Home environment questions

The questions around participants' environments during the home-condition, indicated that most of the 37 participants used a desktop computer (*n* = 24; 56.75%) rather than a laptop (*n* = 13; 35.13%). The most popular browser was Chrome Browser (*n* = 21; 56.75%) followed by Microsoft Edge (*n* = 7; 57%), Explorer (*n* = 1; 0.2%), Internet Explorer (*n* = 1; 0.2%), Mozilla (*n* = 4; 10.8%), and Safari (*n* = 3; %). A total of *n* = 6 of the participants (16.2%) were interrupted, such as by a family member or the doorbell ringing. A further *n* = 2 participants (5.41%) reported technical problems (such as Wi-Fi connectivity issues). Finally, one participant (2.7%) admitted watching/listening a video whilst completing the study.

## Discussion

We set out to develop a version of the short-term memory binding task that is suitable for remote, self-administration. We developed a version of this with user discussions and then trialled the final version with 37 healthy older adults. We found no statistically significant differences regardless of whether participants were tested at home or in the lab. These results were corroborated by further Bayesian Analysis which found no evidence for differences between data collected at home and data collected in the lab. Furthermore, the self-report questions around the home environment indicates whilst some participants were disturbed or experienced technical problems, we still found no difference between home- or lab-testing data.

The data obtained from the home-testing environment questions indicates that most participants experienced no disruptions or technical difficulties while conducting the self-administered test at home. Interestingly, only one participant reported engaging in a secondary task, such as watching or listening to a video, during the home-testing session. While it is conceivable that participants may have provided socially desirable responses, the consistency between the laboratory and home data undermines such a possibility. Nonetheless, without video recording of the at-home assessment, we cannot exclude the possibility that participants were disengaged during testing with secondary activities or recruited family members to assist or indeed take the at-home test.

These results have important opportunities and implications for AD research. In the future, the version of the VSTMB tasks developed here may facilitate the recruitment of large-sampled cohorts to assess if previous effects using the in-person VSTMBT holds across serial testing (i.e. reduce dropouts in longitudinal studies). Moreover, as a valid tool, the online version can be used for baseline and repeated assessments with the latter being highly recommended as it can provide within-subject variability of memory scores.^29,30^ These findings also contribute towards the body of evidence supporting the feasibility of remote neuropsychological assessments for both research and clinical assessment.^31–35^

Crucially, this online version of the task provides the prospect of increasing the diversity of participants who take part in AD research and may mitigate some of the on-going service and research disruptions following the pandemic. Similarly, given the task's simple instructions, low linguistic demands, and educational as well as cultural-insensitivity,^19^ this digital version may offer cost-effective culturally-appropriate measures in low-resourced settings, such as the low-to-middle-income countries.^36^ Nonetheless, we must remain cautiously optimistic about the potential reach of this online web-based tool relative to traditional versions. Indeed, the present study has two primary limitations that should be considered. Firstly, the data were obtained from a relatively small number of healthy older adults (although the study was adequately powered), thereby necessitating the replication of the current findings in older adults with cognitive impairment. Secondly, the sample consisted primarily of well-educated white participants, thereby necessitating the examination of these results in a large-scale study with individuals from diverse demographic backgrounds in future studies. Additionally, the questions around the home environment were not validated, and it is possible we missed some important detail that may have influenced performance. Future and on-going work by our group addresses these shortcomings and will explore barriers of use, such as digital literacy and poverty, which may vary by regional and, indeed, global contexts. Further work will also explore a bench-to-bedside application.

## Supplemental Material

sj-docx-1-dhj-10.1177_20552076241228416 - Supplemental material for The development and validation of a digital biomarker for remote assessment of Alzheimer's diseases risk Click here for additional data file. Supplemental material, sj-docx-1-dhj-10.1177_20552076241228416 for The development and validation of a digital biomarker for remote assessment of Alzheimer's diseases risk by Joe Butler, Tamlyn J Watermeyer, Ellie Matterson and Emily G Harper, Mario Parra-Rodriguez in DIGITAL HEALTH
