# Supplementary material for: The development and validation of a digital biomarker for remote assessment of Alzheimer's diseases risk
Source: Digit Health. 2024 Jan 23;10:20552076241228416. doi: 10.1177/20552076241228416 (PMC10807338; doi:10.1177/20552076241228416)
Supplement: sj-docx-1-dhj-10.1177_20552076241228416 - Supplemental material for The development and validation of a digital biomarker for remote assessment of Alzheimer's diseases risk [file sj-docx-1-dhj-10.1177_20552076241228416.docx]

**Supplementary Materials**

**I. Screening questionnaire (self-reported)**

1. Do you have any visual disturbances, such as colour-blindness? [yes/no]

2. Do you have any physical disability (e.g. major dexterity problems) that would prevent you from participated at a laptop or desktop computer? [yes/no]

3. Have you ever been diagnosed with a learning difficulty that affects your memory or thinking processes (e.g. dementia, ADHD) [yes/no]

**II. Home Environment Questionnaire**

1. I completed this online task using:
2. a laptop
3. a desktop
4. While completing this online task, were you interrupted at any point?
5. Yes
6. No
7. While completing this task did you to music or watch any videos in the background?
8. Yes
9. No
10. During completion of this study, did you experience any technical problems (wifi or technology issues)?
11. Yes
12. No
13. Any other comments that might inform the researchers about the context of your at-home participation:

[free speech box]
